# Scaling-Based Two-Step Reconstruction in Full Polarization-Compressed Hyperspectral Imaging

**DOI:** 10.3390/s20247120

**Published:** 2020-12-11

**Authors:** Axin Fan, Tingfa Xu, Xi Wang, Chang Xu, Yuhan Zhang

**Affiliations:** 1 Key Laboratory of Photoelectronic Imaging Technology and System of Ministry of Education of China, School of Optics and Photonics, Beijing Institute of Technology, Beijing 100081, China; 3120195322@bit.edu.cn (A.F.); 3120185324@bit.edu.cn (X.W.); 3120170324@bit.edu.cn (C.X.); yhzhang@bit.edu.cn (Y.Z.); 2 Beijing Institute of Technology Chongqing Innovation Center, Chongqing 401120, China

**Keywords:** compressive sensing, computational imaging, polarization hyperspectral imaging, two-step reconstruction

## Abstract

Polarized hyperspectral images can reflect the rich physicochemical characteristics of targets. Meanwhile, the contained plentiful information also brings great challenges to signal processing. Although compressive sensing theory provides a good idea for image processing, the simplified compression imaging system has difficulty in reconstructing full polarization information. Focused on this problem, we propose a two-step reconstruction method to handle polarization characteristics of different scales progressively. This paper uses a quarter-wave plate and a liquid crystal tunable filter to achieve full polarization compression and hyperspectral imaging. According to their numerical features, the Stokes parameters and their modulation coefficients are simultaneously scaled. The first Stokes parameter is reconstructed in the first step based on compressive sensing. Then, the last three Stokes parameters with similar order of magnitude are reconstructed in the second step based on previous results. The simulation results show that the two-step reconstruction method improves the reconstruction accuracy by 7.6 dB for the parameters that failed to be reconstructed by the non-optimized method, and reduces the reconstruction time by 8.25 h without losing the high accuracy obtained by the current optimization method. This feature scaling method provides a reference for the fast and high-quality reconstruction of physical quantities with obvious numerical differences.

## 1. Introduction

Due to reflecting the rich spectral and spatial characteristics, hyperspectral imaging is widely used in agricultural detection [1,2], food production [3,4], biomedical identification [5,6] and other fields. Polarization imaging plays an indispensable role in environmental monitoring [7,8], astronomical observation [9,10], tumorigenesis detection [11,12] and other aspects. Meanwhile, polarization hyperspectral imaging naturally shows multiple applications in non-destructive quality evaluation [13,14]. However, according to the initial definition of Stokes parameters [15], it is quite complicated to adjust the polarizers and capture six snapshots in each spectral band. Furthermore, the traditional Fourier Transform Spectropolarimeter (FTSP) [16] not only contains up to two retarders and an analyzer, but also requires a certain time to scan the optical path difference domain and a lot of memory to store the measurement data. In addition, the FTSP cannot flexibly select a specific spectral band of interest. These defects severely limit the application of polarization spectral imaging to fast and low-cost detection of targets.

In order to overcome the defects caused by the FTSP, the compressive sensing (CS) theory [17] has been introduced into the spectropolarimeter. The popular compressive spectral polarization imaging can be divided into several categories according to the measurement principle. Classically, the polarization information can be modulated mainly by several kinds of devices or combinations, for example, two birefringent crystals [18], a micropolarizer array [19,20], a liquid crystal modulator [21], two polarization gratings [22], or a quarter-wave plate (QWP) and a retarder and a polarizer [23]. However, limited by the performance of the modulator, the above compressive imaging techniques [18,19,20,21,22,23] can only obtain the linear Stokes parameters. The lack of circular polarization information has always attracted the attention of many researchers, both in measurement strategies [24] and applications [25]. Recently, the Channeled Compressive Imaging Spectropolarimeter (CCISP) [26] modulates the full-Stokes parameters using the module composed by a polarizer filters wheel, two retarders and a polarizer. Unfortunately, the CCISP system is more complicated than before, and does not provide the selection for the spectral band of interest. The required number of measurements is greater than the number of Stokes parameters obtained.

**Figure 1:**
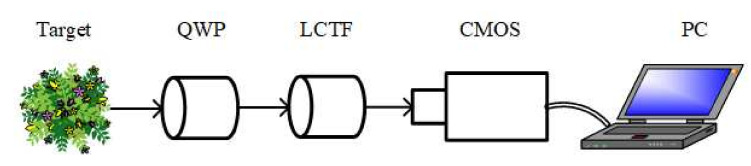
The schematic diagram of FPCHI system.

To avoid the drawbacks of CCISP, we proposed the full polarization-compressed hyperspectral imaging (FPCHI) system. As shown in Figure 1, the FPCHI system consists of only a QWP, a liquid crystal tunable filter (LCTF) and a complementary metal oxide semiconductor (CMOS) detector. The LCTF serves as a combination of spectral filters to provide the selection for the spectral bands of interest. Moreover, by optimizing the sparse basis with machine learning algorithms, we can successfully reconstruct four Stokes parameters with high accuracy through less than four measurements [27]. However, the optimization process added to reduce the number of measurements consumes a lot of time, and the optimized sparse basis may not be suitable for some targets that are not involved in optimization. Although there are many image reconstruction approaches based on CS theory [28,29], none of them can simultaneously achieve the fast and efficient reconstruction of four Stokes parameters.

In this paper, we therefore propose a two-step reconstruction method for the FPCHI system to achieve the fast and efficient reconstruction of the full-Stokes parameters simultaneously under compressive measurement. It is well known that the first Stokes parameter represents the total light intensity, and the last three Stokes parameters represent various light intensity differences. Therefore, the first Stokes parameter and the last three Stokes parameters are not in the same order of magnitude. According to the inherent numerical difference of the four Stokes parameters, the reconstruction process is divided into two steps. Both steps are reconstructed based on the CS theory. Specifically, the first Stokes parameter comes from the first reconstruction, and the last three Stokes parameters come from the second reconstruction. Due to relying on the inherent properties of the four Stokes parameters, the two-step reconstruction method can be used to reconstruct the full-Stokes parameters of any targets. In addition, we verify this proposed method based on the polarized hyperspectral images measured in our laboratory. In order to further illustrate the effectiveness of the two-step reconstruction method, it is also compared with the above optimization method and the non-optimized method.

## 2. Materials and Methods

### 2.1. Full Polarization-Compressed Hyperspectral Imaging

As shown in Figure 1, the FPCHI system is mainly composed of a QWP, an LCTF and a CMOS detector. The QWP combines LCTF to realize the modulation and compression of full polarization information represented by four Stokes parameters. In addition, the LCTF serves as an ideal hyperspectral filter to select the certain spectral band of interest. Then, the CMOS detects the light intensity image after polarization compression in each spectral band.

**Figure 2:**
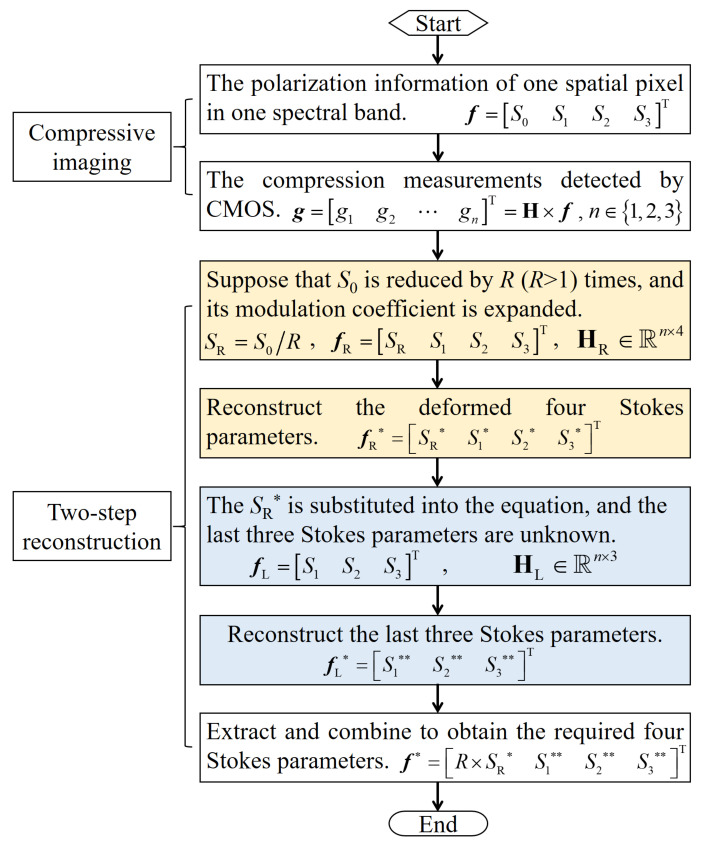
The flowchart of full polarization compression and reconstruction.

Let $F\in R{}^{4\times N_{\lambda }\times N_{x}\times N_{y}}$ represent the polarized hyperspectral images of the target, including four Stokes parameters, $N_{\lambda }$ spectral bands and $N_{x}\times N_{y}$ spatial pixels. The polarization compression for each spectral band is independent, as is for each spatial pixel. Therefore, we consider the polarization compression of one spatial pixel in one spectral band. Let $f={\left[S_{0},\;S_{1},\;S_{2},\;S_{3}\right]}^{T}\in R^{4\times 1}$ represent the polarization information of one spatial pixel in one spectral band. Figure 2 shows the flowchart of full polarization compression and reconstruction.

Full polarization compression can be achieved by the combination of QWP and LCTF. The Mueller matrices of QWP and LCTF are denoted as $M_{1}$ and $M_{2}$ respectively, so the combined Mueller matrix is $M=M_{2}\times M_{1}$, which can be expressed as

(1) $$\begin{array}{c} M=M_{2}\times M_{1}\\ =\frac{1}{2}\left[\begin{array}{cccc} 1 & \cos\left(2\alpha \right) & \sin\left(2\alpha \right) & 0\\ -\cos\left(2\alpha \right) & -\cos^{2}\left(2\alpha \right) & -\cos\left(2\alpha \right)\sin\left(2\alpha \right) & 0\\ -\sin\left(2\alpha \right) & -\cos\left(2\alpha \right)\sin\left(2\alpha \right) & -\sin^{2}\left(2\alpha \right) & 0\\ 0 & 0 & 0 & 0 \end{array}\right]\\ \times \left[\begin{array}{cccc} 1 & 0 & 0 & 0\\ 0 & \cos^{2}\left(2\theta \right) & \cos\left(2\theta \right)\sin\left(2\theta \right) & -\sin\left(2\theta \right)\\ 0 & \cos\left(2\theta \right)\sin\left(2\theta \right) & \sin^{2}\left(2\theta \right) & \cos\left(2\theta \right)\\ 0 & \sin\left(2\theta \right) & -\cos\left(2\theta \right) & 0 \end{array}\right]\\ =\frac{1}{2}\left[\begin{array}{cc} 1 & \cos\left(2\alpha \right)\cos^{2}\left(2\theta \right)+\sin\left(2\alpha \right)\cos\left(2\theta \right)\sin\left(2\theta \right)\\ -\cos\left(2\alpha \right) & -\cos^{2}\left(2\alpha \right)\cos^{2}\left(2\theta \right)-\cos\left(2\alpha \right)\sin\left(2\alpha \right)\cos\left(2\theta \right)\sin\left(2\theta \right)\\ -\sin\left(2\alpha \right) & -\cos\left(2\alpha \right)\sin\left(2\alpha \right)\cos^{2}\left(2\theta \right)-\sin^{2}\left(2\alpha \right)\cos\left(2\theta \right)\sin\left(2\theta \right)\\ 0 & 0 \end{array}\right.-\\ \left.\begin{array}{cc} \cos\left(2\alpha \right)\cos\left(2\theta \right)\sin\left(2\theta \right)+\sin\left(2\alpha \right)\sin^{2}\left(2\theta \right) & -\cos\left(2\alpha \right)\sin\left(2\theta \right)+\sin\left(2\alpha \right)\cos\left(2\theta \right)\\ -\cos^{2}\left(2\alpha \right)\cos\left(2\theta \right)\sin\left(2\theta \right)-\cos\left(2\alpha \right)\sin\left(2\alpha \right)\sin^{2}\left(2\theta \right) & \cos^{2}\left(2\alpha \right)\sin\left(2\theta \right)-\cos\left(2\alpha \right)\sin\left(2\alpha \right)\cos\left(2\theta \right)\\ -\cos\left(2\alpha \right)\sin\left(2\alpha \right)\cos\left(2\theta \right)\sin\left(2\theta \right)-\sin^{2}\left(2\alpha \right)\sin^{2}\left(2\theta \right) & \cos\left(2\alpha \right)\sin\left(2\alpha \right)\sin\left(2\theta \right)-\sin^{2}\left(2\alpha \right)\cos\left(2\theta \right)\\ 0 & 0 \end{array}\right], \end{array}$$

where α(α∈{0,90}) represents the linear polarization angle of the LCTF at the incident plane, and θ(0≤θ<180) denotes the fast axis angle of QWP. By combining the QWP and LCTF, the four Stokes parameters $f_{1}\in R^{4\times 1}$ can be modulated at one time, and $f_{1}=M\times f$. The light intensity information, which is sensitive to the CMOS detector, is only represented by the modulated first Stokes parameter. After one modulation, the first row of the combined Muller matrix **M** makes up the modulation matrix of the proposed system. Moreover, the values of α and θ are required to satisfy that the elements in the first row of matrix **M** are all non-zero values. Thus, after n(n∈N+) modulations, the modulation matrix of this system can be written as

(2) $$\begin{array}{c} H=\frac{1}{2}\left[\begin{array}{cc} 1 & \cos\left(2\alpha_{1}\right)\cos^{2}\left(2\theta_{1}\right)+\sin\left(2\alpha_{1}\right)\cos\left(2\theta_{1}\right)\sin\left(2\theta_{1}\right)\\ 1 & \cos\left(2\alpha_{2}\right)\cos^{2}\left(2\theta_{2}\right)+\sin\left(2\alpha_{2}\right)\cos\left(2\theta_{2}\right)\sin\left(2\theta_{2}\right)\\ \vdots & \vdots \\ 1 & \cos\left(2\alpha_{n}\right)\cos^{2}\left(2\theta_{n}\right)+\sin\left(2\alpha_{n}\right)\cos\left(2\theta_{n}\right)\sin\left(2\theta_{n}\right) \end{array}\right.-\\ \left.\begin{array}{cc} \cos\left(2\alpha_{1}\right)\cos\left(2\theta_{1}\right)\sin\left(2\theta_{1}\right)+\sin\left(2\alpha_{1}\right)\sin^{2}\left(2\theta_{1}\right) & -\cos\left(2\alpha_{1}\right)\sin\left(2\theta_{1}\right)+\sin\left(2\alpha_{1}\right)\cos\left(2\theta_{1}\right)\\ \cos\left(2\alpha_{2}\right)\cos\left(2\theta_{2}\right)\sin\left(2\theta_{2}\right)+\sin\left(2\alpha_{2}\right)\sin^{2}\left(2\theta_{2}\right) & -\cos\left(2\alpha_{2}\right)\sin\left(2\theta_{2}\right)+\sin\left(2\alpha_{2}\right)\cos\left(2\theta_{2}\right)\\ \vdots & \vdots \\ \cos\left(2\alpha_{n}\right)\cos\left(2\theta_{n}\right)\sin\left(2\theta_{n}\right)+\sin\left(2\alpha_{n}\right)\sin^{2}\left(2\theta_{n}\right) & -\cos\left(2\alpha_{n}\right)\sin\left(2\theta_{n}\right)+\sin\left(2\alpha_{n}\right)\cos\left(2\theta_{n}\right) \end{array}\right]. \end{array}$$

In order to realize the compression measurement of the four Stokes parameters, *n* is less than four. Then, the n(n∈{1,2,3}) compression measurements detected by CMOS can be denoted as $g={\left[g_{1},\;g_{2},\;\cdots ,\;g_{n}\right]}^{T}\in R^{n\times 1}$, and

(3) g=H×f.

The compression measurements of each spatial pixel in each spectral band are combined to form the full polarization-compressed hyperspectral images $G\in R^{n\times N_{\lambda }\times N_{x}\times N_{y}}$. For the actual experiment, **G** is a known matrix obtained by CMOS, and **H** is determined by the current system parameters. In particular, **F** is an unknown matrix to be reconstructed.

### 2.2. Scaling-Based Two-Step Reconstruction

Inspired by polarization compression, we consider the polarization reconstruction of one spatial pixel in one spectral band. Since the first Stokes parameter is much larger than the other three Stokes parameters, it is difficult to reconstruct the four Stokes parameters at the same time. In this paper, the four Stokes parameters are reconstructed in two steps using feature scaling.

In the first step, suppose that the first Stokes parameter $S_{0}$ is reduced by R(R>1) times to obtain a new parameter $S_{R}$, that is, $S_{R}=S_{0}/R$. Let $f_{R}={\left[S_{R},\;S_{1},\;S_{2},\;S_{3}\right]}^{T}\in R^{4\times 1}$ represent the deformed polarization information. Meanwhile, the modulation coefficients corresponding to the first Stokes parameter are expanded by *R* times to obtain a new modulation matrix as follows:

(4) $$\begin{array}{c} H_{R}=\frac{1}{2}\left[\begin{array}{cc} R & \cos\left(2\alpha_{1}\right)\cos^{2}\left(2\theta_{1}\right)+\sin\left(2\alpha_{1}\right)\cos\left(2\theta_{1}\right)\sin\left(2\theta_{1}\right)\\ R & \cos\left(2\alpha_{2}\right)\cos^{2}\left(2\theta_{2}\right)+\sin\left(2\alpha_{2}\right)\cos\left(2\theta_{2}\right)\sin\left(2\theta_{2}\right)\\ \vdots & \vdots \\ R & \cos\left(2\alpha_{n}\right)\cos^{2}\left(2\theta_{n}\right)+\sin\left(2\alpha_{n}\right)\cos\left(2\theta_{n}\right)\sin\left(2\theta_{n}\right) \end{array}\right.-\\ \left.\begin{array}{cc} \cos\left(2\alpha_{1}\right)\cos\left(2\theta_{1}\right)\sin\left(2\theta_{1}\right)+\sin\left(2\alpha_{1}\right)\sin^{2}\left(2\theta_{1}\right) & -\cos\left(2\alpha_{1}\right)\sin\left(2\theta_{1}\right)+\sin\left(2\alpha_{1}\right)\cos\left(2\theta_{1}\right)\\ \cos\left(2\alpha_{2}\right)\cos\left(2\theta_{2}\right)\sin\left(2\theta_{2}\right)+\sin\left(2\alpha_{2}\right)\sin^{2}\left(2\theta_{2}\right) & -\cos\left(2\alpha_{2}\right)\sin\left(2\theta_{2}\right)+\sin\left(2\alpha_{2}\right)\cos\left(2\theta_{2}\right)\\ \vdots & \vdots \\ \cos\left(2\alpha_{n}\right)\cos\left(2\theta_{n}\right)\sin\left(2\theta_{n}\right)+\sin\left(2\alpha_{n}\right)\sin^{2}\left(2\theta_{n}\right) & -\cos\left(2\alpha_{n}\right)\sin\left(2\theta_{n}\right)+\sin\left(2\alpha_{n}\right)\cos\left(2\theta_{n}\right) \end{array}\right]. \end{array}$$

Therefore, Equation (3) can be rewritten as

(5) $$g=H_{R}\times f_{R}.$$

Then, the deformed polarization information is reconstructed based on the CS theory. The deformed polarization information is first sparsely represented by

(6) $$f_{R}=\Psi_{R}\times \theta_{R}.$$

In this equation, $\Psi_{R}$ is a known sparse basis matrix, and $\theta_{R}$ is the sparse coefficient vector to be reconstructed. Substituting Equation (6) into Equation (5), we can obtain that

(7) $$g=H_{R}\times \Psi_{R}\times \theta_{R}=A_{R}\times \theta_{R},$$

where $A_{R}$ represents the sensing matrix of this system, which can be calculated from $A_{R}=H_{R}\times \Psi_{R}$. Based on the two-step iterative shrinkage/thresholding (TwIST) algorithm [30], the sparse coefficient vector $\theta_{R}$ can be reconstructed by solving the optimization problem as follows:

(8) $${\hat{\theta }}_{R}=\arg\min_{\theta_{R}}{∥g-A_{R}\times \theta_{R}∥}_{2}^{2}+\tau_{R}{∥\theta_{R}∥}_{1},$$

where $\tau_{R}$ is the weight of $l_{1}$-norm regularization. In this ill-posed problem, normally a priori can be considered as two types, Laplace distribution and Gaussian distribution. The $l_{1}$-norm regularization is suitable for solving parameters that obey the Laplace prior distribution, and the $l_{2}$-norm regularization is suitable for solving parameters that obey the Gaussian prior distribution. In addition, the commonly used $l_{1}$-norm is applied as the regularization term in Equation (8) because the $l_{1}$-norm can obtain a sparse solution. Substituting the reconstructed sparse coefficient vector into Equation (6), we can obtain the deformed polarization information, denoted as ${f_{R}}^{*}={\left[{S_{R}}^{*},\;{S_{1}}^{*},\;{S_{2}}^{*},\;{S_{3}}^{*}\right]}^{T}$.

In the second step, the last three Stokes parameters are reconstructed again. Substituting the deformed first Stokes parameter into Equation (5), we can obtain the equation containing only the last three Stokes parameters as

(9) $$g-1/2\times R\times {S_{R}}^{*}=H_{L}\times f_{L}.$$

In Equation (9), $H_{L}\in R^{n\times 3}$ is composed of columns 2 to 4 of matrix $H_{R}$ in Equation (4), and $f_{L}={\left[S_{1},\;S_{2},\;S_{3}\right]}^{T}\in R^{3\times 1}$. Then, the last three Stokes parameters can also be reconstructed based on CS theory. First, the last three Stokes parameters can be sparsely represented by

(10) $$f_{L}=\Psi_{L}\times \theta_{L},$$

where $\Psi_{L}$ is the known sparse basis matrix, and $\theta_{L}$ is the unknown sparse coefficient vector. By substituting Equation (10) into Equation (9), the new equation can be obtained as

(11) $$g-1/2\times R\times {S_{R}}^{*}=H_{L}\times \Psi_{L}\times \theta_{L}=A_{L}\times \theta_{L}.$$

Using the TwIST algorithm [30] again, the sparse coefficient vector $\theta_{L}$ can be reconstructed by solving the following optimization problem:

(12) $${\hat{\theta }}_{L}=\arg\min_{\theta_{L}}{∥(g-1/2\times R\times {S_{R}}^{*})-A_{L}\times \theta_{L}∥}_{2}^{2}+\tau_{L}{∥\theta_{L}∥}_{1}.$$

Then according to Equation (10), the last three Stokes parameters can be calculated and denoted as ${f_{L}}^{*}={\left[{S_{1}}^{**},\;{S_{2}}^{**},\;{S_{3}}^{**}\right]}^{T}$.

Finally, the required four Stokes parameters can be obtained by combining the reconstruction results from the above two steps. The last three Stokes parameters are taken from the reconstruction results of the second step. The first Stokes parameter is obtained by deforming the reconstruction result of the first step. According to the scaling rule in the first step, the first Stokes parameter is *R* times the deformed parameter. In other words, let $f^{*}$ represent the required four Stokes parameters, then $f^{*}={\left[R\times {S_{R}}^{*},\;{S_{1}}^{**},\;{S_{2}}^{**},\;{S_{3}}^{**}\right]}^{T}$. The reconstructed four Stokes parameters of each spatial pixel in each spectral band are combined to form the polarized hyperspectral images of the target $F^{*}\in R{}^{4\times N_{\lambda }\times N_{x}\times N_{y}}$.

## 3. Results

### 3.1. Acquisition of Polarized Hyperspectral Images for FPCHI Input

**Figure 3:**
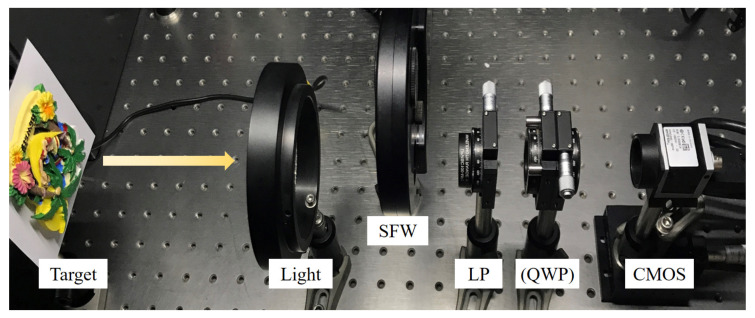
The definition-based polarization hyperspectral imaging (DBPHI) system.

In order to measure the polarized hyperspectral images as the simulation input of the FPCHI system, we first established the definition-based polarization hyperspectral imaging (DBPHI) system [15]. As shown in Figure 3, the DBPHI system mainly consists of a spectral filters wheel (SFW), a linear polarizer (LP), a QWP and a CMOS. The SFW includes a series of narrow-band spectral filters used to select the spectral band of interest. For each spectral filter, the LP serves to measure the linear polarization states, and the LP and QWP are combined to measure the circular polarization states. Then, the four Stokes parameters can be calculated from

(13) $$\left.\begin{array}{c} S_{0}=I_{0}+I_{90}\\ S_{1}=I_{0}-I_{90}\\ \;\;\;S_{2}=I_{45}-I_{135}\\ S_{3}=I_{R}-I_{L} \end{array}\right\},$$

where the linear polarization states $I_{0},\;I_{90},\;I_{45}$ and $I_{135}$ can be obtained by sequentially rotating the polarization angle of LP to 0, 90, 45 and 135. Moreover, the right-hand circular polarization state $I_{R}$ can be measured through the LP with −45 polarization angle and the QWP with horizontal fast axis. The left-hand circular polarization state $I_{L}$ can be achieved through the LP with +45 polarization angle and the QWP with horizontal fast axis.

**Figure 4:**
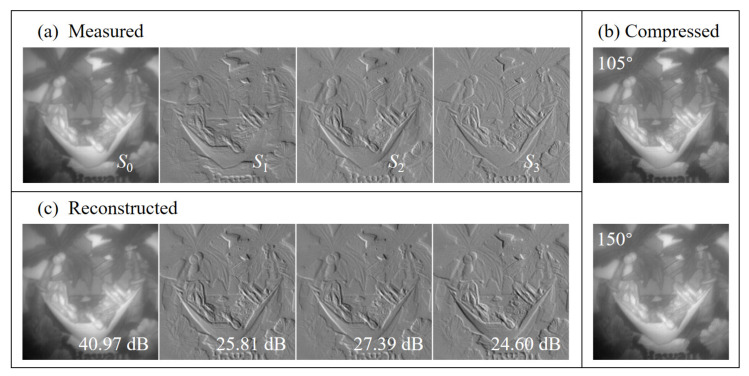
The (**a**) measured, (**b**) compressed and (**c**) reconstructed images in the spectral band of 600 nm.

In the experiment, the SFW is equipped with 18 spectral filters (Thorlabs, Newton, USA; FB520-10, FB530-10, …, FB690-10) whose center wavelengths are from 520 nm to 690 nm with an interval of 10 nm. The LP (Thorlabs, Dachau, Germany; LPVISC100-MP2) works effectively from 510 nm to 800 nm, and the QWP (Thorlabs, Newton, USA; SAQWP05M-700) operates from 325 nm to 1100 nm. The CMOS detector (Basler, Ahrensburg, Germany; acA2040-180 km) has 1024×1024 pixels in the spatial domain. Through the above operations and calculations, the measured polarized hyperspectral images are denoted as $F\in R^{4\times 18\times 800\times 800}$, including four Stokes parameters, 18 spectral bands and 800×800 spatial pixels. The four Stokes parameters images measured in the spectral band of 600 nm are shown in Figure 4a.

### 3.2. Compressive Imaging and Two-Step Reconstruction of FPCHI System

Then, full polarization compression and reconstruction are simulated on the measured images of each spatial pixel in each spectral band. Assume that polarization modulation is performed twice. The linear polarization angle of the incident surface of LCTF remains 0, and the fast axis angles of QWP are 105 and 150, respectively. That is, $n=2,\;\alpha_{1}=\alpha_{2}=0,\;\theta_{1}=105,\;\theta_{2}=150$. Substituting these parameters into Equation (2), we can obtain the modulation matrix of this system as follows:

(14) $$H=\left[\begin{array}{cccc} 0.5 & 0.375 & 0.2165 & 0.25\\ 0.5 & 0.125 & -0.2165 & 0.4330 \end{array}\right].$$

**Figure 5:**
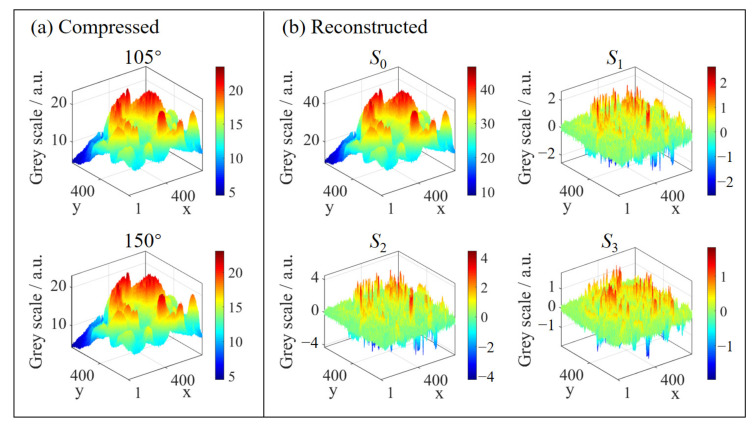
The grey distribution of (**a**) compressed images and (**b**) reconstructed polarized images in the spectral band of 600 nm.

Therefore, full polarization-compressed hyperspectral images $G\in R^{2\times 18\times 800\times 800}$ can be obtained through the modulation matrix. Figure 4b shows the polarization-compressed images in the spectral band of 600 nm. The grayscale distribution of the compressed images is shown in Figure 5a.

As described in Section 2.2, the full polarization information is reconstructed in two steps. In the first step, suppose that the first Stokes parameter $S_{0}$ is reduced by 100 times to obtain a new parameter $S_{R}$, that is, $S_{R}=S_{0}/100$. Thus, the modulation coefficients corresponding to the first Stokes parameter are increased by 100 times, and the new modulation matrix is

(15) $$H_{R}=\left[\begin{array}{cccc} 50 & 0.375 & 0.2165 & 0.25\\ 50 & 0.125 & -0.2165 & 0.4330 \end{array}\right].$$

The discrete W transform (DWT) matrix [31] severs as the sparse basis of four Stokes parameters $\Psi_{R}\in R^{4\times 4}$, which is expressed as

(16) $$\Psi_{R}=\left[\begin{array}{cccc} 0.6533 & 0.6533 & 0.2706 & -0.2706\\ 0.6533 & -0.6533 & 0.2706 & 0.2706\\ 0.2706 & 0.2706 & -0.6533 & 0.6533\\ -0.2706 & 0.2706 & 0.6533 & 0.6533 \end{array}\right].$$

Based on Equations (6)–(8), we can reconstruct the deformed polarization information ${f_{R}}^{*}={\left[{S_{R}}^{*},\;{S_{1}}^{*},\;{S_{2}}^{*},\;{S_{3}}^{*}\right]}^{T}$. In the Equation (8), $\tau_{R}$ is 0.005.

In the second step, the modulation matrix corresponding to the last three Stokes parameters is

(17) $$H_{L}=\left[\begin{array}{ccc} 0.375 & 0.2165 & 0.25\\ 0.125 & -0.2165 & 0.4330 \end{array}\right].$$

The DWT matrix [31] is also used as the sparse basis of the last three Stokes parameters $\Psi_{L}\in R^{3\times 3}$, which is expressed as

(18) $$\Psi_{L}=\left[\begin{array}{ccc} 0.7887 & 0.5774 & -0.2113\\ 0.5774 & -0.5774 & 0.5774\\ -0.2113 & 0.5774 & 0.7887 \end{array}\right].$$

Then according to Equations (10)–(12), these three parameters can be reconstructed ${f_{L}}^{*}={\left[{S_{1}}^{**},\;{S_{2}}^{**},\;{S_{3}}^{**}\right]}^{T}$. Similarly in the Equation (12), $\tau_{L}$ is 0.005.

By integrating the reconstruction results based on these two independent steps, we can obtain the ideal four Stokes parameters $f^{*}={\left[100\times {S_{R}}^{*},\;{S_{1}}^{**},\;{S_{2}}^{**},\;{S_{3}}^{**}\right]}^{T}$. Finally, the polarized hyperspectral images can be reconstructed as $F^{*}\in R^{4\times 18\times 800\times 800}$. As the example illustrated in Figure 4c, the four Stokes parameters images reconstructed in the spectral band of 600 nm achieve good recovery effect. Meanwhile, Figure 5b shows the grayscale distribution of the reconstructed polarized images.

**Figure 6:**
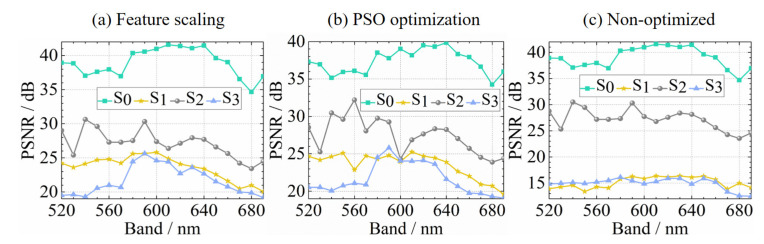
The PSNR values of polarized hyperspectral images reconstructed with (**a**) feature scaling, (**b**) the sparse basis optimized by PSO, and (**c**) non-optimized method.

In order to compare with feature scaling, we also reconstruct the polarized hyperspectral images not only with the sparse basis optimized by particle swarm optimization (PSO) [27], but also with non-optimized method. Using the measured image as a reference, the peak signal to noise ratio (PSNR) value of each reconstructed image is calculated for all three kinds methods. In the Figure 6, the three sub-images (a), (b) and (c) show the PSNR values of polarized hyperspectral images reconstructed by the proposed feature scaling method, the sparse basis optimized by PSO method and the non-optimized method, respectively.

## 4. Discussion

It can be seen from Equation (13) that the first Stokes parameter represents the total light intensity, and the last three Stokes parameters represent the light intensity differences. Specifically, $S_{1}$ and $S_{2}$ represent the light intensity differences of linear polarization states, and $S_{3}$ represents the light intensity difference of circular polarization states. Therefore, the first Stokes parameter is much larger than the last three Stokes parameters, resulting in the failure to display in the same grayscale range. As shown in Figure 4a, the four Stokes parameters are well displayed in their respective grayscale ranges. In addition, it can be seen from Figure 4b that there is no obvious difference between the two polarization-compressed images. This is because the compressed image mainly contains the information of the first Stokes parameter, which is determined by both the numerical characteristics of the first Stokes parameter and the modulation matrix of the system represented by Equation (14). As Figure 4c shows, the texture feature of each Stokes parameter has been well reconstructed by the proposed two-step reconstruction method, although the last three Stokes parameters account for very little in the compressed image.

Furthermore, Figure 5 more clearly reflects the grayscale distribution of the images numerically. As described in the previous analysis of Figure 4b, the grayscale distribution of the compressed images in Figure 5a is very similar. The grayscale distribution of the compressed image under the fast axis angle of 105 is between 4.47 and 23.45, and that under the fast axis angle of 150 is between 4.47 and 23.10. Then, in conjunction with the reconstructed images in Figure 5b, it can be seen that the shape of the grayscale distribution of the first Stokes parameter image is similar to that of the compressed images, while their grayscale ranges are different. The grayscale range of the first Stokes parameter image is from 8.96 to 46.54, which is about twice over that of the compressed image. This numerical relationship is consistent with the polarization modulation matrix of the system. Finally, Figure 5b also reflects that the grayscale distribution of the last three Stokes parameters is one order of magnitude smaller than that of the first Stokes parameter. Moreover, the last three Stokes parameters all contain negative values. These phenomena can be explained by the definition of the Stokes parameters expressed in Equation (13). At the same time, this further illustrates both the difficulty of full polarization reconstruction and the effectiveness of the two-step reconstruction method.

The reconstruction effect can be seen not only from the image details, but also from the PSNR values shown in Figure 6. For each Stokes parameter, take the average of the PSNR values in 18 spectral bands. For the three reconstruction methods shown in Figure 6, the average PSNR values of the four Stokes parameters are shown in Table 1. Obviously, the reconstruction results of feature scaling and PSO optimization are better than those of non-optimized method. However, the sparse basis optimized by PSO may not be able to reconstruct the polarized images of any target so well. In addition, the feature scaling reconstruction takes 1.65 h, while the PSO optimization and reconstruction require time consuming as high as 9.9 h. Therefore, the feature scaling performs better than PSO, and has the potential to be widely used.

In particular, there are two reasons why the two-step reconstruction method can quickly and accurately reconstruct polarization information. On the one hand, the first Stokes parameter contributes much more to the compressed image than the last three Stokes parameters. As a result, when the four Stokes parameters are reconstructed at the same time, the weight flooding problem makes the reconstruction of the last three Stokes parameters seriously affected by the first Stokes parameter. The two-step reconstruction method solves this problem. In the first step where all four Stokes parameters are involved, only the reconstruction result of the first Stokes parameter is retained. Then, the last three Stokes parameters are reconstructed in the second step, avoiding the interference of the first Stokes parameter. On the other hand, the reconstruction error can be better controlled under the two-step reconstruction method. In the two steps, different reconstruction algorithms can be used or the regularization weight value can be adjusted to accurately improve the reconstruction accuracy of each Stokes parameter. Moreover, there is no need to learn to optimize the sparse basis, which not only greatly improves the reconstruction efficiency, but also is widely applicable to various targets with different polarization characteristics.

## 5. Conclusions

The FPCHI system can achieve both compression measurement of full-Stokes parameters and hyperspectral imaging. The QWP with adjustable fast axis angle unites the LCTF with selectable linear polarization angle to modulate the four Stokes parameters. Meanwhile, the LCTF also enables flexible selection among a large number of fine spectral bands to meet hyperspectral imaging. Then for each spectral band, the two-step reconstruction method can recover four Stokes parameters from the compressed measurements based on feature scaling. In the two steps, the first Stokes parameter is extracted from the first reconstruction, and the last three Stokes parameters are from the second reconstruction.

Based on the polarized hyperspectral images measured in our laboratory, both the polarization-compressed hyperspectral imaging of the FPCHI system and the two-step reconstruction method are simulated sequentially. The simulation results perform well in the evaluation of image display, grayscale distribution and PSNR values. Compared with the optimization method using machine learning, the reconstruction accuracy of the two-step reconstruction method does not decrease and still maintains a satisfactory level. Further in terms of reconstruction efficiency, the two-step reconstruction method shortens the 9.9 h required for the optimization method to 1.65 h, which means a time reduction of 83.33%.

The grayscale value of the first Stokes parameter is quite different from the last three Stokes parameters, so the two-step reconstruction method separates their reconstruction processes. This method is conducive to more accurately reduce the reconstruction error of each step. Since it is based on the inherent properties of the Stokes parameters, this method is suitable for various targets with different polarization characteristics. Moreover, for the compression of the spatial domain or other domains, this provides a reference for the reconstruction of parameters that are originally at different orders of magnitude. However, this approach still faces some limitations. Next, we will focus on solving the intelligent and appropriate selection of feature scaling values. Through further optimization, such as optimizing the polarization encoding pattern, more efficient reconstruction will be achieved to apply the polarization hyperspectral imaging to actual target detection and other monitoring fields.

## Figures and Tables

**Table 1:** The average PSNR values of the four Stokes parameters reconstructed based on feature scaling, PSO optimization and Non-optimized.

Reconstruction Method	$S_{0}$ / dB	$S_{1}$/dB	$S_{2}$/dB	$S_{3}$/dB
Feature scaling	38.98	23.59	27.12	21.67
PSO optimization	37.35	23.52	27.45	21.67
Non-optimized	38.97	15.14	27.21	14.91

## Abbreviations

The following abbreviations are used in this manuscript:

FTSP	Fourier Transform Spectropolarimeter
CS	compressive sensing
QWP	quarter-wave plate
CCISP	Channeled Compressive Imaging Spectropolarimeter
FPCHI	full polarization-compressed hyperspectral imaging
LCTF	liquid crystal tunable filter
CMOS	complementary metal oxide semiconductor
TwIST	two-step iterative shrinkage/thresholding
DBPHI	definition-based polarization hyperspectral imaging
SFW	spectral filters wheel
LP	linear polarizer
DWT	discrete W transform
PSO	particle swarm optimization
PSNR	peak signal to noise ratio
